# Angiomatoid fibrous histiocytoma occurring at distal/acral extremity sites: clinicopathological and molecular study of 26 cases highlighting frequent myxoid histology and site-dependent genotypic variation

**DOI:** 10.1007/s00428-025-04199-y

**Published:** 2025-08-01

**Authors:** Abbas Agaimy, Jeremy Molligan, Fatimah I. Alruwaii, Cristina R. Antonescu, Elizabeth G. Demicco, Brendan C. Dickson, John Gross, Michael Michal, Kyle Perry, Lars Tögel, Robert Stoehr, Nasir Ud Din, Andrew L. Folpe

**Affiliations:** 1 Institute of Pathology, Friedrich-Alexander University Erlangen-Nürnberg (FAU), University Hospital Erlangen (UKER), Erlangen, Krankenhausstraße 8-10, 91054 Germany; 2 Comprehensive Cancer Center Erlangen-EMN (CCC ER-EMN), Erlangen, Germany; 3 Department of Laboratory Medicine and Pathology, Mayo Clinic, Rochester, MN USA; 4 Department of Pathology and Laboratory Medicine, Henry Ford Health System, Detroit, MI USA; 5 Department of Pathology, Memorial Sloan-Kettering Cancer Center, New York, NY USA; 6 Department of Pathology and Laboratory Medicine, Mount Sinai Hospital and Laboratory Medicine and Pathobiology, University of Toronto, Toronto, Canada; 7 Department of Pathology, John Hopkins University, Baltimore, MD USA; 8 Department of Pathology, Faculty of Medicine in Plzen, Charles University, Plzen, Czech Republic; 9 Bioptical Laboratory, Ltd, Plzen, Czech Republic; 10 Department of Pathology, University of Michigan, Ann Arbor, MI USA; 11 Department of Pathology and Laboratory Medicine, University of Miami Miller School of Medicine, Miami, FL USA

**Keywords:** Targeted next generation sequencing, Clear cell sarcoma, Precision medicine, Genetic landscape, Profiling, Mimics

## Abstract

Angiomatoid fibrous histiocytoma (AFH) is a rare mesenchymal neoplasm of borderline malignancy (locally recurring, rarely metastasizing), most often involving the limbs, trunk, and head/neck. Rarely, AFH may involve unusual locations. Herein, we characterize the clinicopathologic features of 26 AFH of the distal extremities, including acral sites. The tumors occurred in 19 females and 7 males ranging in age from 12 to 76 years (median, 23 years). Tumors involved the upper (*n* = 19) and lower (*n* = 6) distal extremity; one affected an unspecified digital site. Twenty-two cases occurred in acral locations (hands and feet). Subsets of cases showed the following morphologic features: multinodular architecture (26/26), lymphoid cuffs (23/26), prominent stromal myxoid change (11/25), angiomatoid features (9/26), and cytologic pleomorphism (8/26). The average mitotic count was 1/10 HPF; 3 cases showed brisk mitotic activity (> 10 mitoses/10 HPF). Immunohistochemistry revealed variable expression of desmin (16/25), EMA (14/21) and ALK (5/8). Molecular testing revealed *EWSR1* rearrangements in 17/18 cases (94%). Among 12 tumors with known fusion partners, the fusions partner was *CREB1* in 6 cases (50%), *CREM* in 4 tumors (33%), *ATF1* in one tumor (8%) and *PBX3* (8%) in another tumor. Prominent myxoid features were noted in 75% *CREM* versus 33% of *CREB1* versus 0% of *ATF1*-fused tumors. AFH occurring in distal extremity/acral locations have a predilection for females, upper extremity locations, frequent unusual (solid, non-angiomatoid and myxoid) morphology and higher frequency of *CREM* over *ATF1* fusions. Awareness of the morphologic spectrum of these rare neoplasms is essential for correct classification.

## Introduction

Angiomatoid fibrous histiocytoma is a rare low-grade mesenchymal neoplasm of unknown histogenesis accounting for < 0.3% of all soft tissue neoplasms and is characterized by intermediate biology with occasional local recurrences and low metastatic potential [1, 2]. The superficial (subcutaneous) soft tissues of the extremities are the most frequently affected sites followed by the trunk and the head and neck regions [1, 2]. Rarely, deep lesions and unusual locations (visceral sites, lungs, retroperitoneum, mediastinum, and intra-abdominal sites) have been reported as well [3, 4], but the nosology of some reported cases at these unusual sites is controversial. Males and females are affected similarly at a wide age range, but most are in the range of 10–20 years [1, 2]. Remarkably, a proportion of patients with detailed clinical histories and/or preoperative serologies may show paraneoplastic symptoms and signs resulting probably from excessive IL-6 secretion by the neoplastic cells and causing elevated C-reactive protein (CRP), unexplained fever, anemia, thrombocytosis and other related symptoms [5]. These symptoms usually resolve completely after tumor resection [5].

Histologically the prototypical AFH consists of epithelioid or ovoid to spindle cells arranged in a syncytial pattern, forming multiple nodules/lobules within a variably fibrous stroma. Prominent cystic and hemorrhagic pseudovascular spaces are characteristic, frequently mimicking hemangioma, hematoma, or a reactive lesion clinically [1, 2]. Up to 2/3 of cases occur at sites of lymph nodes (inguinal, axillary, popliteal, cubital, and the neck) and the neoplasms are frequently surrounded by a circular lymphoid cuff, closely mimicking a lymph node at low power examination. However, true intranodal origin has not been convincingly documented.

Having encountered rare AFH cases at acral sites that represented a diagnostic challenge due to unusual morphology, we aimed in this study to characterize a large series of AFH originating at distal or acral extremity sites to gain more insight into their morphological and genotypic spectrum and to address, whether they recapitulate AFH in general or might display phenotypic or genetic distinctness.

## Material and methods

The cases were identified in the consultation files of the authors. The tissue specimens were fixed in formalin and processed routinely for histopathology. All tumors were diagnosed as AFH with or without molecular profiling by expert soft tissue pathologists. The histological slides and the reports were reviewed and the key features of AFH assessed for each case: lobulation/nodularity, fibrous/fibromyxoid versus myxoid stroma, angiomatoid features, cellular pleomorphism, mitotic activity and lymphoid cuffs. Immunoreactivity for desmin, EMA and ALK was recorded if available. Molecular testing has been performed at different institutions and using different methods/platforms (details of the staining protocols, antibody sources and the molecular methods used are available upon request).

## Results

### Clinical features

Patients were 17 females and 9 males with an age range of 12–76 years (median, 23; Table 1). Eight patients (31%) were pediatric (< 18 years). The median age was 17 and 27 for males and females, respectively. The clinical presentation was painless swelling in most cases, with a clinical impression of a benign lesion such as ulcerated fibroma/non-healing ulcer, ganglion cyst, hemangioma, or vascular malformation in 9 cases. Two cases were thought to be possible tenosynovial giant cell tumors and another two as schwannoma or subungual exostosis.

**Table 1:** Clinicopathological and molecular findings in distal/acral angiomatoid fibrous histiocytomas (*n* = 26)

No	Age/sex	Site/size cm	Lobulated/multinodular	Lymphoid cuff	Angiomatoid	Pleomorphic cells	Mitoses/10 hpfs	Stroma	ALK	Desmin	EMA	Gene fusion	Clinical features/impression
1	40/F	L hand/NA	+	-	-	-	< 2	Myxoid	+	-	-	EWSR1::CREB1	“Hand wound”
2	52/F	D4 L hand/NA	+	+ F	-	-	0	Myxoid	+	+	+	EWSR1::CREB1	NA
3	18/M	Planter R/NA	+	+	-	+	> 10	Fibromyxoid	-	-	+	EWSR1::CREB1	“Non-healing ulcer”
4	14/M	5th metacarpal/NA	+	-	-	-	< 1	Myxoid	+	-	+	EWSR1::CREM	NA
5	60/M	L index finger/NA	-	+	+	-	1	Sparse fibrous	+ wk	-	+	EWSR1::PBX3	NA
6	12/F	Wrist/NA	+	+	+	+	17	-	NA	+	-	NA	Hemangioma?
7	35/F	Foot/NA	-	+	+	+	< 2	-	NA	+	+ F	Failed	Recurrent
8	19/F	Hand – interdigital/NA	+	+	-	-	1	Myxoid	NA	-	+	NA	Mass
9	23/F	Wrist/2.9	-	+	-	-	0	Sparse fibrous	NA	+	-	NA	Giant cell tumor, Fibroma?
10	15/M	Wrist/1.7	+	+	-	-	1	Myxoid	NA	+	-	EWSR1 FISH +	Clinically fibroma/ganglion
11	26/F	Hand (palm)/NA	+	-	-	-	0	Fibrous	NA	NA	NA	EWSR1 FISH +	Mass
12	21/F	Hand (palm)/NA	+	+	+	-	2	Fibrous/sclerotic	NA	-	-	EWSR1::ATF1	Mass
13	28/F	R ring finger/1.5	+	+	_	_	0	Fibrous/sclerotic	NA	+	+ F	EWSR1::CREB1 EWSR1 FISH +	“Vascular malformation”
14	29/F	R distal hallux/NA	+	+	-	-	0	Sparse/fibrous	-	+	+	NA	Subungual exostosis
15	17/M	R thumb/NA	+	+	+	-	1	Fibrous	NA	-	-	EWSR1/FUS FISH-	Mass
16	13/F	R foot/NA	+	+	+	-	6	Fibromyxoid	NA	NA	NA	NA	“phlebolith”
17	16/F	R wrist/NA	+	+	-	+	1	Fibrous	NA	+	+	NA	“Schwannoma”
18	24/M	R palm/NA	+	+	-	+	1	Myxoid	NA	+	+	EWSR1 FISH +	“ulcerated fibroma/abscess”
19	21/M	R long finger/NA	+	+	-	-	2	Myxoid	NA	+	-	NA	“EMC”
20	15/M	R great toe/NA	+	+	-	-	10	Myxoid	NA	+ (rare)	+	EWSR1 FISH +	“Traumatized fibroma/recurrence”
21	23/F	L thumb/NA	+	+	-	+	1	Myxoid	NA	+	NA	EWSR1 FISH +	Giant cell tumor
22	76/F	L ring finger/1.8	+	+	+	+	0	Fibromyxoid	NA	+	NA	EWSR1::CREB1	Clinically ganglion cyst
23	28/F	2./3.metacarpal/NA	+	+	-	-	0	Myxoid	-	+ F	+	EWSR1::CREM	NA
24	69/F	R D1/4.9	+	+	+	-	0	Myxoid	NA	+ F	+	EWSR1::CREM	Lesion for 7–8 yrs
25	13/M	R middle finger/NA	+	+	+	+	2	Fibrous/sclerotic	NA	-	NA	EWSR1::CREB1	NA
26	27/F	Ankle/NA	+	+	-	-	> 10/10	NA	+ F	+ F	+	EWSR1::CREM	NA

*F*, focal; *NA*, not available; *L*, left; *R*, right

**Fig. 1:**
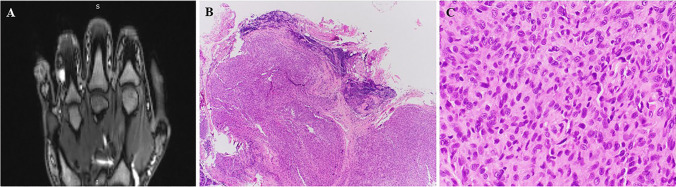
Example of conventional AFH of distal extremity/acral sites. (**A**) Imaging showed contrast-enhancing small subcutaneous nodule on the 4th finger. (**B**) Lower power view of same lesion showed well-circumscribed lobulated cellular neoplasm with prominent peripheral lymphoid cuffs and homogeneous cellularity (note absence of angiomatoid features). (**C**) High power showing monomorphic epithelioid to plump elongated cells disposed into syncytial solid nodular growth (images from Case 13 with *EWSR1::CREB1* fusion)

Size was available for 5 cases with a range of 1.5–4.9 cm (median, 1.8). The upper extremity (hand including the fingers, palmar surface, and wrist joint) was the most frequent site accounting for 20 cases (80%) (Fig. 1A). Five tumors affected the foot including the ankle joint area. One tumor involved an unspecified digit. Notably, the digits were the site of origin in 10 cases (8 in the fingers, one in a toe and one at unspecified digit). Of 16 cases with known laterality, 11 were on the right side compared to 5 lesions on the left extremity.

### Pathological findings

**Fig. 2:**
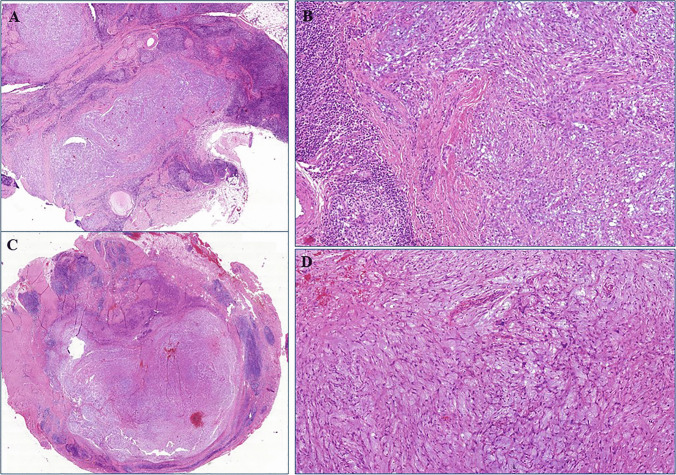
(**A**) This lesion showed prominent myxoid lobules surrounded by extensive lymphoid aggregates. (**B**) Higher magnification shows “fasciitis-like” loose myxoid spindle cell lesion surrounded by lymphoid cuffs. (**C** + **D**) Another case with very similar low- and high-power appearance as depicted in A and B (A + B = Case 8; C + D = Case 10)

**Fig. 3:**
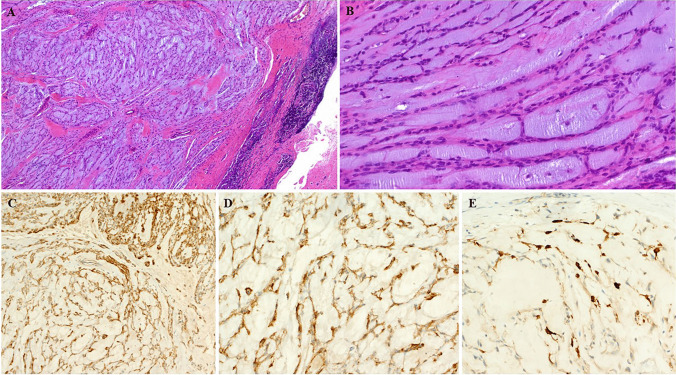
This case (Case 23 with *EWSR1::CREM* fusion) showed prominent lobulated reticular-myxoid pattern with prominent lymphoid cuffs (**A**) and chordoid arrangements of the neoplastic cells with in myxohyaline stroma reminiscent of extraskeletal myxoid chondrosarcoma (**B**). Immunohistochemistry of same case showing diffuse expression of EMA (**C**), ALK (**D**) and patchy reactivity with desmin (**E**)

**Fig. 4:**
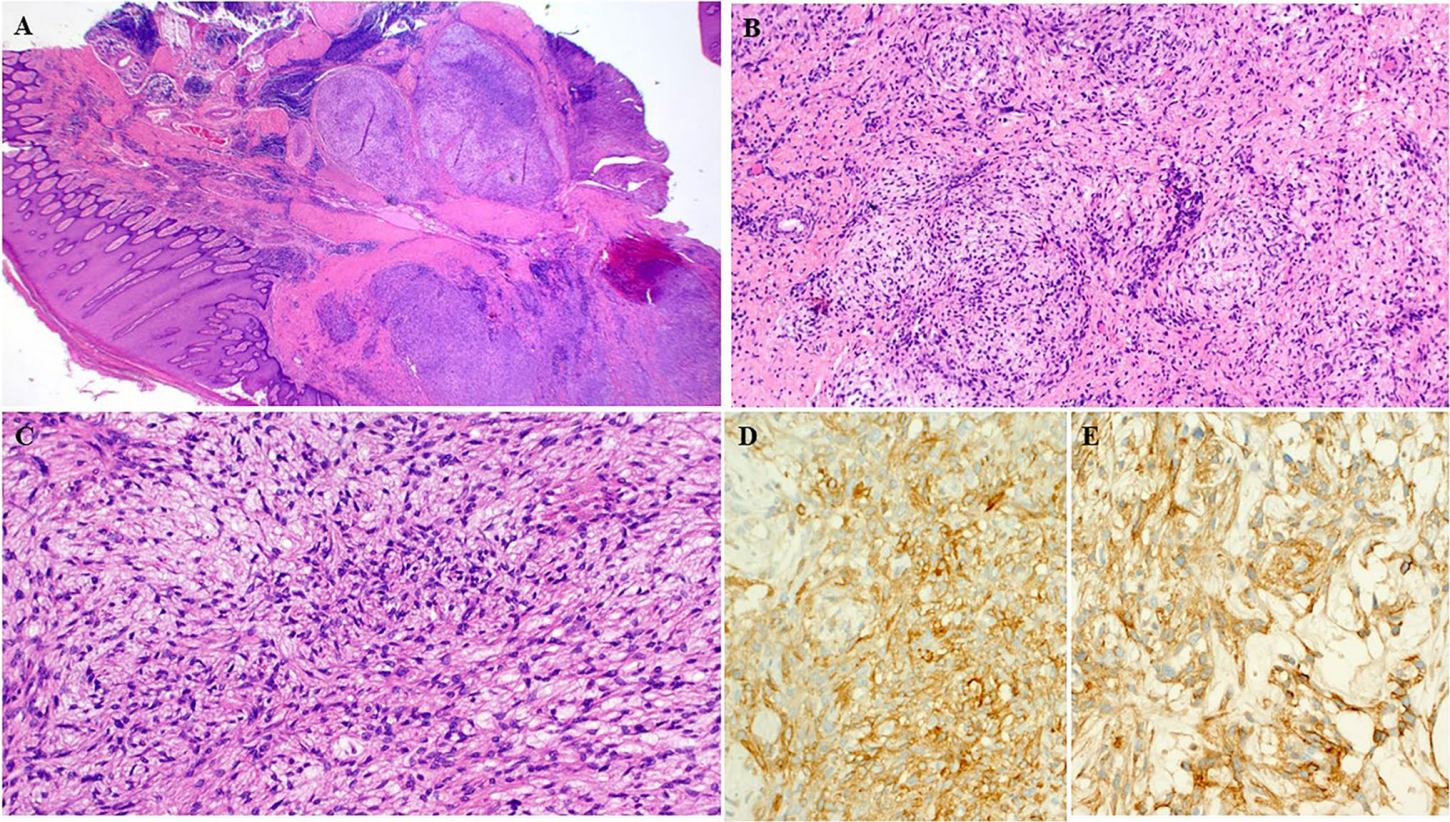
This dermal plantar tumor (Case 3 with *EWSR1:CREB1* fusion) showed prominent irregular lobulation with prominent lymphoid cuffs but no angiomatoid features (**A**). (**B**) myxoid fibrous whorls with neuroid features represent a diagnostic pitfall. (**C**) Angiomyxoid/fibromyxoid features at this anatomic site might be mistaken for acral fibromyxoma. (**D**) expression of ALK may be misinterpreted as ALK-associated myxoid dermal neoplasm. (**E**) diffuse EMA reactivity

**Fig. 5:**
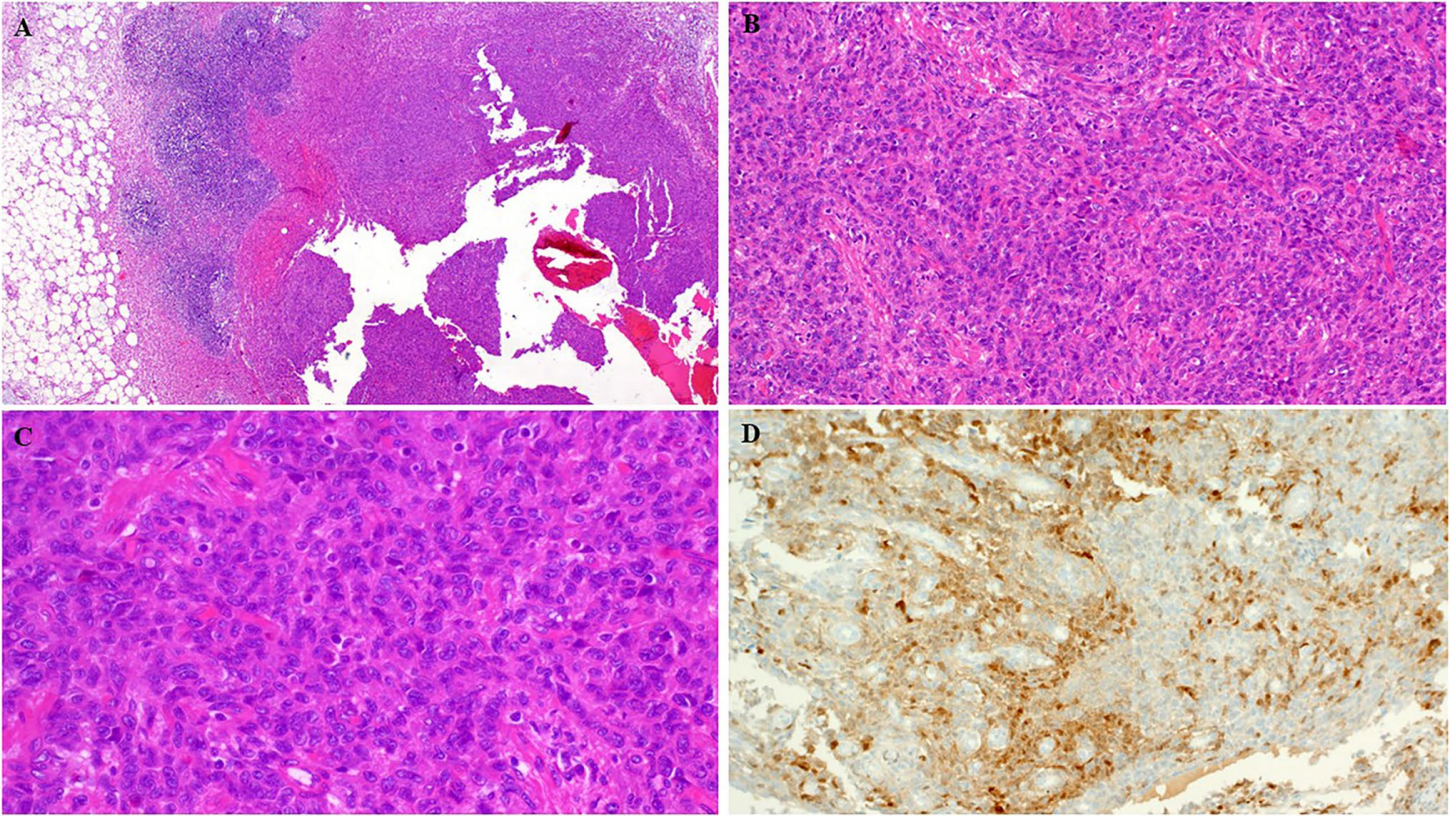
This case (Case 5 with *EWSR1::PBX3* fusion) showed characteristic features of AFH at low power with prominent lymphoid cuffs and angiomatoid features within the subcutaneous fat (**A**). Intermediate (**B**) and high (**C**) power showed monotonous ovoid and epithelioid cells arranged into diffuse solid sheets. Testing for markers of syncytial myoepithelioma revealed patchy expression of S100 (**D**), but no smooth muscle actin or p63 reactivity

All tumors were superficially located. Prominent lobulation with variable multinodularity at low-power examination was present in 23 of 26 cases (88%). Peripheral lymphoid cuffs were evident in 23 cases (88%), being prominent in 21 cases and focally present in two tumors (Fig. 1B). Only 9 cases (35%) had angiomatoid or hemorrhagic features. Scattered cells showing more than mild cellular pleomorphism were noted in 8 cases (31%). The mitotic counts ranged from 0 to 17 mitoses per 10 HPFs (median, 1). The stromal characteristics were purely and prominently myxoid in > 60% of the tumor areas in 11 (42%), sparsely fibrous to sclerotic in 12 (46%) and fibromyxoid in 3 (12%) of cases. Representative examples of the morphological features are shown in Figs. 1, 2, 3, 4, 5.

Immunohistochemistry was notable for variable expression of EMA in 14/21 (67%; Fig. 3C), ALK in 5 of 8 (63%; Fig. 3D) and desmin in 16/25 (64%; Fig. 3E), with four tumors showing limited desmin reactivity and the remainder more diffuse expression.

### Molecular genetic findings

Overall, molecular genetic testing was performed in 19 cases: one failed due to poor RNA quality. Eighteen cases were successfully tested either by targeted RNA sequencing (11 cases) or by FISH probes targeting *EWSR1*, *FUS*, *CREB1* or *ATF1* gene loci (7 cases). *EWSR1* rearrangements were detected in 17 of the 18 cases (94%). Both fusions partners were known in 12 tumors. In these, *CREB1* was the fusion partner in 6 cases (50%), while 4 tumors (33%) harbored *CREM* fusions. One tumor each had an *EWSR1::ATF1* (8%) and *EWSR1::PBX3* (8%) fusion.

### Phenotype-genotype correlations

Prominent myxoid features were noted in 2 of 6 *EWSR1::CREB1* positive tumors (33%), in 3 of 4 *EWSR1::CREM* positive tumors (75%), but not in the single cases with *EWSR1::ATF1* or *EWSR1::PBX3* fusions. Regarding anatomic sites, 5 of the 6 *CREB1*-positive cases were in the hand/fingers. Two of the 4 *CREM* positive cases were located in the metacarpal region of the hand, one in the ankle and one at unspecified digit.

The *EWSR1::PBX3* fused case showed typical histological features of AFH (prominent lymphoid cuffs, angiomatoid changed and expressed EMA and ALK). This case was tested in addition with markers frequently expressed in syncytial myoepithelioma and revealed patchy expression of S100 but was negative with smooth muscle actin and p63.

## Discussion

Fusions between the *FET* gene family (*EWSR1* and *FUS*) and one of the *CREB* transcription factor family members (*ATF*, *CREB1*, and *CREM*) have been increasingly reported in a variety of mesenchymal neoplasms that are clinically, anatomically, and phenotypically distinct [6–8]. Among these, *EWSR1::CREB1/ATF1/CREM* fusions have been recognized as the most prevalent drivers in clear cell sarcoma of tendons and aponeuroses (CCS) [9], malignant gastrointestinal neuroectodermal tumor (MGNET) [10], angiomatoid fibrous histiocytoma (AFH) [11–13], primary pulmonary myxoid sarcoma [14] as well as in emerging categories of epithelioid, mostly keratin-positive intra-abdominal neoplasms/sarcomas [15] and in primary intracranial myxoid sarcomas not fitting any specific category [16]. The latter may resemble extra-skeletal myxoid chondrosarcoma.

Several reports have described myxoid variants of *EWSR1::CREB* fused neoplasms in soft tissue and intrathoracic location. For some, the terminology “myxoid variant of AFH” has been applied, based on their unequivocal similarity to conventional AFH of soft tissue, irrespective of their unusual or unexpected anatomic site [3, 4]. In general, however, prominent myxoid features in AFH are uncommon and may pose diagnostic challenges, especially when these tumors occur at unusual sites such as the distal extremities or acral sites.

While the exact site (proximal versus distal) was not detailed in many large series of AFH, 24% and 8% of AFH in the upper and lower extremities have been reported to originate at distal sites, respectively [1, 2]. However, acral locations have not been specified in these series. Schaefer and Fletcher reported 21 myxoid AFH cases defined by a myxoid pattern in > 60% of the tumors [17]. Their series revealed a slight predominance of females (62%), in line with our present data, which was 73% female. Six of the 21 (29%) cases reported by Schaefer and Fletcher were located at distal/acral sites (5 in the hand/fingers and one ankle lesion). However, only limited genetic data was available in that study, with *EWSR1* rearrangements being detected by FISH in 4 of 7 cases, but fusion partners were unknown.

In this study, we tried to gain insight into the clinicopathological features and genetic characteristic of AFH at distal extremities and acral sites to address whether these tumors are different from conventional AFH occurring at more frequent proximal extremity sites. Our study shows that distally/acrally located AFH tend to affect females more frequently than males with a higher median age for females (27 versus 17 years). Moreover, a diffuse myxoid pattern is overrepresented in acrally/distally located extremity AFH (42% of cases). Our findings contrast with the general anatomic distributions of AFH in prior larger unselected series where the upper extremities were affected in 43–51% of cases (versus 80% upper extremity location in our series).

AFH is characterized by recurrent *EWSR1::CREB1* fusions resulting from the t(2;22)(q33;q12) in > 90% of cases [11–13]. However, involvement of other *CREB* gene family members as fusions partners have been increasingly recognized, including *EWSR1::ATF1* and *EWSR1::CREM* fusions [7, 11–13]. While there is no clear phenotype-genotype correlation in AFH, it has been proposed that extrasomatic cases tend to be enriched for the *EWSR1::ATF1* fusion type [11–13]. In this study, we found that acrally/distally located AFH are characterized by a somewhat different distribution of genotypes, as compared to AFH in general [11–13]. Notably, 33% of our cases with identified fusion partners harbored *EWSR1::CREM* fusions compared to only a single case with *EWSR1::ATF1* fusion (8%). *EWSR1::ATF1* fusion was once considered the second most frequent fusion type after *EWSR1::CREB1* in AFH [11–13]. Accordingly, our results suggest that *CREM* fusion variants likely are enriched among distally/acrally located AFH cases with a frequency of 33%, compared to unselected AFH cohorts in the literature, where *CREB1* and *ATF1* fusions are detected in > 90% of cases [11–13]. Moreover, 75% of our *CREM* fusion cases are predominantly or diffusely myxoid compared to 29% myxoid pattern frequency in tumors harboring the *CREB1/ATF1* fusions. Finally, we report a novel *EWSR1::PBX3* fusion in one case. This fusion characterizes the vast majority of syncytial myoepithelioma of cutaneous and non-cutaneous origin including malignant myoepithelial tumors of bone [18, 19]. The *EWSR1::PBX3* fused AFH case showed typical features of AFH including prominent lymphoid cuffs and angiomatoid changed and expressed EMA and ALK. This case was tested in addition with markers frequently expressed in syncytial myoepithelioma and revealed patchy expression of S100 but no smooth muscle actin or p63 reactivity. Accordingly, it remains unclear whether this particular case represents genetic variant in the spectrum of AFH (as evidenced by classical AFH morphology) or, alternatively, a morphological pattern in the spectrum of syncytial myoepithelioma.

While the diagnosis of conventional AFH is straight-forward for experienced soft tissue pathologists, morphologically unusual tumors may be considerably more challenging [20]. These include tumors with solid, pure spindle cell [21], pleomorphic [22], reticular-angiomatoid [23], or schwannoma-like features, as well as those containing osteoclast-like giant cells, a “myoepithelioma-like” reticular-myxoid pattern [20], and “osteosarcoma-like” calcifying variants in bone [24]. In general, these unusual morphologic variants tend to lack angiomatoid spaces and a lymphoid cuff, compounding these difficulties.

The differential diagnosis of acrally or distally located myxoid AFH is limited but can be challenging. It encompasses variably myxoid entities including acral fibromyxomas, extraskeletal myxoid chondrosarcoma, ganglion cyst with mucus extravasation, ALK + epithelioid cell histiocytoma, ALK-rearranged myxoid dermal neoplasms, ALK-positive fusion-driven rhabdomyosarcoma and other myxoid lesions.

The presence of myxoid features and ALK immunoreactivity might be mistaken for an ALK-rearranged neoplasm, particularly the recently described ALK-rearranged superficial myxoid neoplasms [25]. These rare lesions affect mostly adults at a mean age of 51 years. Most occur at non-distal sites (back, thigh, knee, and shin). Histologically, they are distinct as well with spindled and ovoid cells arranged in cords and concentric whorls within a myxoid to myxohyaline stroma. They lack prominent lymphocytic aggregates or lymphoid cuffs. Notably, they uniformly express ALK and CD34 and are almost always positive for S100 protein but are EMA-negative. Molecular genetic profiling has shown *ALK* fusions in all cases studied with *FLNA, MYH10*, and *HMBOX1* as main fusion partners [25].

In summary, we have described 26 angiomatoid fibrous histiocytomas presenting at distal extremity and acral sites. Our study highlights interesting anatomic-phenotypic-genotypic correlations for AFH in these locations, in particular myxoid change and *CREM* fusions. The morphological diversity of these rare tumors at these unusual sites may pose diagnostic challenges, such that they may be mistaken for a variety of other site-specific entities or reactive lesions.

## Data Availability

The datasets generated during and/or analyzed during the current study are not publicly available, but are available from the corresponding author upon reasonable request.
